# Prevalence and Clinical Significance of Early Repolarization in Athletes: A Systematic Review

**DOI:** 10.1111/anec.70032

**Published:** 2024-12-08

**Authors:** Khaled Elenizi

**Affiliations:** ^1^ Department of Internal Medicine, College of Medicine Prince Sattam Bin Abdulaziz University Alkharj Saudi Arabia

**Keywords:** athletes, early repolarization, electrocardiography (ECG), preparticipation screening, ST‐segment elevation, sudden cardiac death

## Abstract

**Introduction:**

Early repolarization (ER) is an electrocardiographic pattern characterized by J‐point and ST‐segment elevation, frequently observed in athletes. Initially deemed benign, recent studies suggest a possible association between ER and increased risks of cardiac arrhythmias and sudden cardiac death, necessitating a thorough examination of its clinical implications in athletes.

**Methods:**

A comprehensive literature review was conducted using MEDLINE (via PubMed) and EMBASE databases, focusing on articles related to ER in athletes. Search terms included “early repolarization,” and relevant studies were selected based on their focus on athletic populations. A total of 22 articles were included for detailed analysis.

**Results:**

The review encompassed 22 studies with a combined total of 44,326 athletes, revealing an overall mean ER prevalence of 31.6 ± 17.6 (*p* < 0.001). Most common location in the inferolateral region at 32.28%. The prevalence varied significantly across studies, ranging from 7% to 89%, influenced by factors such as age, gender distribution, and athletic discipline. Male athletes exhibited a higher incidence of ER compared with females, and endurance athletes showed a greater prevalence than strength athletes.

**Conclusion:**

ER is notably prevalent among athletes, especially males and those engaged in endurance sports. Current studies do not establish a direct association between ER and increased mortality in athletes. Further research is essential to refine risk stratification criteria and develop appropriate management strategies to ensure athlete safety while maintaining optimal performance levels.

## Abstract 2

Early repolarization (ER) is common in athletes, with a 31.6% prevalence, especially among males and endurance athletes. Although no direct link to increased mortality was found, ER's association with cardiac arrhythmias warrants further research to better understand risk factors and develop management strategies for athlete safety.

**Abbreviations**
ACC: American College of Cardiology
AHA: American Heart Association
bpm: beats per minute
CAD: coronary artery disease
CI: confidence interval
CV: cardiovascular
ECG: electrocardiogram
ER: early repolarization
HCM: hypertrophic cardiomyopathy
HRS: Heart Rhythm Society
NA: not available
OR: odds ratio
SCD: sudden cardiac death
SCI: spinal cord injuries
STE: ST‐segment elevation
ST‐segment: ST segment
VF: ventricular fibrillation

## 1 Introduction

In 1936, Shipley and Hallaran first described early repolarization (ER) in young adults (Shipley and Halloran 1936). They considered the J‐wave at the end of the QRS complex as a normal variant in healthy individuals. Since then, this finding has been regarded as a benign variant commonly observed in young, athletic, or physically active individuals and was defined as ST elevation without chest pain. (Wasserburger and Alt 1961; Klatsky et al. 2003) However, in 2008, Haïssaguerre et al. reported their findings on ER and its association with an increased risk of sudden cardiac arrest (SCA). Over time, further research has explored the potential link between ER and an elevated risk of cardiac arrhythmias and sudden cardiac death in specific individuals (Haïssaguerre et al. 2008; Tikkanen et al. 2009; Junttila et al. 2012).

Cardiovascular‐related sudden death is the leading cause of mortality in athletes during sport and exercise (Harmon et al. 2011), yet while the association between ER and SCA is known, there is still a significant gap in understanding ER's impact on mortality in athletes. ER, which is common in healthy, young athletes and presents in varying patterns on ECGs—from minor J‐point elevation to pronounced J‐waves and ST‐segment elevation, resembling ischemia or pericarditis—remains a topic of debate, as its role in predicting SCA is unclear. This highlights the need for further research to determine whether ER in athletes is benign or a potential marker for SCA, crucial for improving risk assessment, prevention, and intervention strategies.

This review aims to provide the current understanding of ER in athletes, emphasizing its prevalence, characteristic ECG patterns, and potential implications for arrhythmogenesis and risk stratification. By exploring the interplay between athletic conditioning, autonomic regulation, and ER manifestations, this research seeks to elucidate the clinical significance of ER in athletes and inform appropriate management strategies. The ultimate goal was to ensure both optimal athletic performance and cardiovascular safety.

## 2 Method

A thorough literature search was performed using MEDLINE (via PubMed) and EMBASE to locate original articles, guideline statements, and review papers pertinent to our topic. The search included terms such as “early repolarization.” I included only those that provided sufficient information to estimate the prevalence of ER, focusing on cohort studies and case–control studies published as original articles. In instances where multiple reports on the same population existed, the most recent or informative data were used. Additionally, references within these articles were carefully examined to ensure a complete review of all relevant literature.

## 3 Result

Searches on Embase and PubMed identified 289 and 455 articles, respectively. For Embase, the terms “early repolarization”/exp. OR “early repolarization syndrome”/exp. OR “early repolarization pattern”/exp. were used, while PubMed was searched with early repolarization, early repolarization syndrome, and early repolarization pattern. After the initial screening, studies not focusing on athletic populations were excluded, leaving a smaller pool of relevant articles. Ultimately, 22 articles met the inclusion criteria and were included in the review (see Table 1).

**TABLE 1:**
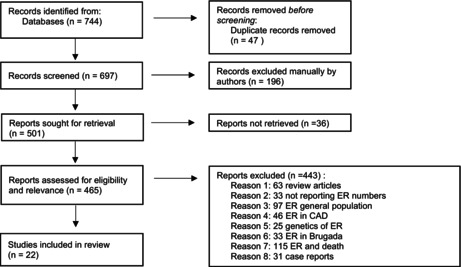
Prisma flow diagram.

The dataset comprises 22 studies, encompassing a total of 44,326 athletes (see Table 2). The mean prevalence of ER in the athletes at 31.6% ± 17.6 (see Table 3). The overall percentage of male athletes is 77.61%. Notably, studies that included only male athletes reported a relatively higher prevalence of ER, with an estimated average of 42.1% across these studies. The average age of athletes varies significantly across studies, ranging from 11.7 to 51 years, indicating a focus on different age groups and athletic levels, from youth to senior athletes. The proportion of male athletes also varies widely, with some studies consisting entirely of male participants, while others have a lower male representation, such as 51% in Juhani Junttila's 2010 study. This variation suggests differing focuses on male versus female athletes across the studies.

**TABLE 2:** Studies included with their results.

Author	Year	# Athletes	Mean age (years)	Men %	ER #	ER %	Criteria	F/U mean years	Sports type and volume	Outcome
Pelliccia et al. (2000)	2000	1005	24	75	144	24	STE > 2 mm	3.4	38 sporting disciplines.	1 HCM developed AFib, no CV event
Bianco et al. (2001)	2001	155	30.9	100	139	89	STE > 1 mm	NA	Runners, soccer, cyclist	NA
Crouse et al. 2009	2007	77	18	100	26	33.8	NR	4	Football	NA
Pelliccia et al. (2007)	2007	32,652	17	80	2280	7	J‐point > 0.1 mm	NA	Soccer, basketball, swimming, cycling	NA
Wilhelm et al. (2010)	2010	54	24	100	25	46	J‐point > 0.1 mm	1	Soccer	NA
Junttila et al. (2011)	2010	503	NA	51	62	30	J‐point > 0.1 mm	10	Preparticipation	No CV event, several SVTs
Noseworthy et al. (2011)	2011	879	18.4	62	221	35.1	J‐point > 0.1 mm	2	Rowers and soccer	No CV event
Kervio et al. (2013)	2012	282	24.4	100	37	13.1	STE > 2 mm	NA	Soccer	No CV event
Aagaard et al. (2013)	2012	153	51	100	49	32	STE > 1 mm	NA	Runners	NA
Muramoto et al. (2014)	2014	1114	19.2	56.7	456	41	STE > 1 mm	7	Preparticipation	No CV event
Brosnan et al. (2014)	2014	1381	20	78.9	109	7.8	J‐point > 0.1 mm	NA	Cycling, Rowing, Swimming, skating, running	No CV event
Quattrini et al. (2014)	2014	704	25	62	102	14	NR	6	30 sporting disciplines.	No CV event
Peidro et al. (2014)	2014	210	18	100	86	40.9	J‐point > 0.1 mm	5	Soccer	No CV event
Aagaard et al. (2014)	2014	151	50.9	100	67	44	STE > 2 mm	NA	Runners	NA
Serra‐Grima et al. (2015)	2015	299	20	66	94	31.4	J‐point > 0.1 mm	24	Athletics, swimming, basketball	No CV event
Konopka et al. (2016)	2016	117	17.5	54.7	35	29.9	J‐point > 0.1 mm	NA	Rowers	NA
Ahmed et al. (2017)	2017	575	15	64	228	40	STE > 1 mm	NA	Basketball, soccer, track, football	NA
De Asmundis et al. (2014)	2017	121	13.5	100	43	36	J‐point > 0.1 mm	1	Soccer	No CV event
Claessen et al. (2020)	2020	2090	42	71	502	24	J‐point > 0.1 mm	3	Static and dynamic	No CV event
Halasz et al. (2021)	2021	886	11.7	72.5	117	13.2	J‐point > 0.1 mm	4.2	17 sporting disciplines	No CV and arrhythmic event
Albiński et al. (2021)	2021	891	14.8	65	280	31.5	STE > 1 mm	NA	45 sporting disciplines	NA
Zimmermann et al. (2022)	2022	27	26.5	100	12	44.4	J‐point > 0.1 mm	NA	Basketball	NA

**TABLE 3:** Mean and standard deviation of ER prevalence in athlete.

	Number of studies	Total number	Mean prevalence	*p*
Athletes	22	44,326	31.6 ± 17.6	*p* < 0.001

The ER percentage varies significantly across studies, ranging from as low as 7% in Antonio Pelliccia's 2007 study, which had the largest number of participants at 32,652, to as high as 89% in M. Bianco's 2001 study. This wide range suggests differences in the populations studied or the criteria used to define ER. For example, Stephen F. Crouse's 2007 study reported a high ER percentage (33.8%) among a small cohort of 77 athletes, suggesting a high odds group or intense monitoring of ER. Femke M.A.P. Claessen's 2020 study of 2090 athletes with a mean age of 42 shows an ER percentage of 24%, focusing on older athletes who might be at higher odds for ER. There does not seem to be a clear trend in ER percentages or mean ages from 2000 to 2022, suggesting that differences are likely due to each study's specific focus and methodology rather than changes over time. Overall, the data show a diverse range of studies with varying focuses on age, gender distribution, and ER rates. Further analysis is needed to understand the specific contexts and reasons behind these variations.

There are nine studies not reporting for follow‐up duration and cardiovascular outcomes, these studies are either retrospective or cross‐sectional in design. As a result, they were unable to provide data on long‐term cardiovascular events, limiting the ability to assess the potential risks associated over time.

### 3.1 Most Common ER Location on ECG for Athletes

Out of the 22 studies reviewed, only 11 provided detailed descriptions of ER location and morphology (see Table 4). On average, ER was most frequently observed in the inferolateral region at 32.28%, followed by lateral only at 21.87%, and Inferior only at 19.73%. The prevalence of J‐waves notch and QRS slurring was comparable, at 29.6% and 24.3%, respectively. The data overall indicate significant variability in the distribution of ER morphology across different studies. Some researchers focused on specific characteristics such as ST‐segment elevation or QRS slurring, while others reported a broader spectrum of ER patterns across various ECG leads. This diversity highlights the complexity of ER interpretation and underscores the importance of standardized reporting to deepen our understanding of this electrocardiographic phenomenon.

**TABLE 4:** ER location and type.

Author	Lateral ER %	Inferior ER %	Infeolateral ER %	ER with J‐wave notch%	ER with QRS slurring %	ER with STE
Matthias Wilhelm	33	13	NR	NR	NR	NR
M. Juhani Junttila	21	20	11	NR	NR	NR
Peter A. Noseworthy	NR	14.9	NR	47	53	NR
David Muramoto	29.9	11.1	NR	8.7	10.4	21.9
Filippo M. Quattrini	25	3	72	12	4	84
PHILIP AAGAARD	NR	NR	NR	44.7	NR	25.3
Ricard Serra‐Grima	57.4	6.4	36.1	15	15	NR
Marcin Konopka	2.8	45.7	51.4	57.1	32.4	11.4
Humera Ahmed	10	42	48	23	31	NR
Carlo De Asmundis	8.2	9.1	18.2	NR	NR	NR
Geza Halasz	9.5	27.3	53.8	NR	NR	NR

## 4 Discussion

### 4.1 Variability in ER Definitions

The term J‐waves was first described and its defining criteria have remained mostly unchanged, with some studies reporting it as ST elevation and others as J‐point elevation. Some studies require the presence of notching or slurring, while others do not. The differences in prevalence of ER among studies are believed to reflect variations in the definition, methods of diagnosis, and differences in the studied population. Some authors consider the QRS complex to include the J‐wave/slurring and locate the J‐point at the onset of the ST‐segment, while others place the J‐point at the intersection between the slur/J‐wave onset and the descending limb of the R‐wave (Maury and Rollin 2013).

Macfarlane et al. define ER as a notch or slur at the end of a prominent R‐wave, positioned entirely above the baseline, J‐point elevation should be ≥ 0.1 mV in two or more adjacent leads of the 12‐lead ECG, except for V1–V3, and QRS duration should be less than 120 ms. (Macfarlane et al. 2015) The 2017 international consensus statement defines ER as a QRS‐ST junction (J‐point) elevation of ≥ 0.1 mV, typically accompanied by a late QRS slurring or notching (J‐wave) impacting the inferior and/or lateral leads (Drezner et al. 2017).

Defining ER is challenging due to its dynamic characteristics, as it can appear and disappear depending on factors like heart rate, autonomic tone, and body position. This variability is especially evident in athletes, who have higher vagal tone. For example, research by Claessen FMAP et al. found that athletes had a 50% higher prevalence of ER compared with a control group (OR 1.5 (SE 0.34), adjusted 95% CI 1.0 to 2.4) based on multivariable analysis. Additionally, Noseworthy, P. A. et al. discovered that exercise training significantly increased the prevalence of ER. These fluctuations make it difficult to establish a consistent criterion for accurately detecting its presence (Rollin et al. 2013).

### 4.2 Differentiating Benign and Malignant ER Patterns

Risk levels associated with ER patterns vary significantly. Benign ER features a J‐point elevation of less than 0.1 mV, a concave upward ST‐segment, stability over time, and no symptoms like syncope. In contrast, malignant ER includes a J‐point elevation greater than 0.2 mV, often in inferior and/or lateral leads, a horizontal or downsloping ST‐segment (Tikkanen et al. 2009), fragmented QRS complexes (Haïssaguerre et al. 2008), multiple regions (Rosso et al. 2008), and potential links to symptoms or a family history of sudden cardiac death. (Tikkanen et al. 2009; Tikkanen et al. 2009; Yonezu et al. 2021; Conte et al. 2016; Kataoka et al. 2016; Gourraud et al. 2013; Haïssaguerre et al. 2008) However, existing definitions often overlook this critical difference, categorizing diverse risk profiles under a singular term (Rosso et al. 2008).

According to the ESC 2022 guidelines, ER is defined by a J‐point elevation of ≥ 1 mm in at least two adjacent inferior or lateral ECG leads, without syncope or resuscitation. ER syndrome is diagnosed in individuals resuscitated from unexplained ventricular fibrillation or polymorphic ventricular tachycardia. ER is common among athletes, appearing in over two‐thirds of cases, prompting concerns about risks. While the AHA, ACC, and HRS consider ER a normal variant, distinguishing between benign and malignant forms is crucial for assessing cardiac event risk (Macfarlane et al. 2015; Rautaharju, Surawicz, and Gettes 2009).

### 4.3 Ethnic Differences in Cardiac Repolarization Patterns Among Athletes

Ethnicity significantly influences cardiac adaptation, with Black athletes showing a notable prevalence of repolarization changes. A study found that ER is present in up to 46% of Black adolescents. (Ahmed et al. 2017) Additionally, more than two‐thirds of Black athletes display J‐point and ST‐segment elevation in leads V1–V4. (Papadakis et al. 2011; Sheikh et al. 2014) These findings are typically considered normal unless accompanied by indications of cardiomyopathy. Understanding these ethnic differences is crucial for accurate ECG interpretation and differentiating between benign and pathological conditions.

Studies in this review consistently shows that ER is more common in Black athletes compared to their white counterparts. For example, in one study 38.9% of Black athletes show ER compared to 21.7% of White athletes (Crouse et al. 2009), with some studies reporting ER rates as high as 63%–91% among Black athletes. (Papadakis et al. 2009; Papadakis et al. 2011) Among a sample of 2090 athletes and 151 nonathletes, ER was notably more prevalent in Black athletes, with an odds ratio (OR) of 1.5 (95% CI: 1.0–2.1, *p* = 0.042). When focusing specifically on lateral leads, the OR was even higher at 2.0 (95% CI: 1.2–3.6, *p* = 0.015). (Claessen et al. 2020). Additionally, the presence of any component of a lateral lead J‐wave pattern was found in 40.5% of African Americans compared to 23.5% of non‐African Americans (*p* < 0.001). (Muramoto et al. 2014) In a cohort of 879 collegiate athletes, ERP was significantly associated with Black race, showing an odds ratio of 5.84 (95% CI: 3.54 to 9.61; *p* < 0.001). (Noseworthy et al. 2011) Among 503 athletes, ER was slightly more common in African Americans (34%) compared with non‐African Americans (28%), although this difference was not statistically significant (*p* = 0.22). (Junttila et al. 2011) Some studies have not reported significant differences in ECG patterns among Black athletes, highlighting the complexity and variability of these findings.

### 4.4 Early Repolarization and Sudden Cardiac Death in Athletes

The annual incidence of sudden cardiac death (SCD) in athletes is relatively low, ranging from 1 in 75,000 to 1 in 200,000. (Corrado et al. 1998; Maron 2003) ER has drawn attention due to its association with arrhythmias and SCD, particularly following Haïssaguerre's study. Since ER is more prevalent in athletes, it has raised special concerns about its potential risks. However, follow‐up periods in studies range from 3 to 24 years, consistently showing no significant cardiovascular events or deaths related to ER in these athlete populations. Furthermore, Cappato et al. found that athletes who had experienced a cardiac arrest did not show recurrent ventricular arrhythmias after stopping sports activities for 3 years, even though early repolarization remained visible on their ECG (Cappato et al. 2010).

Among 704 athletes with 102 having ER, no cardiac events, ventricular tachyarrhythmias, or structural cardiovascular disease were reported over a 6‐year follow‐up. (Quattrini et al. 2014) Similarly, Muramoto's study of 1114 multiethnic athletes found no association between J‐waves, slurs, or ST elevation and increased cardiovascular death risk after 3 years. (Muramoto et al. 2014) Among 879 collegiate athletes with 221 having ER, there were no cases of sudden cardiac death (SCD), unexplained syncope, or cardiovascular hospitalizations. (Noseworthy et al. 2011) In a cohort of 299 white elite athletes with 31.4% having ER, no SCD episodes occurred after 24 years. (Serra‐Grima et al. 2015) A study of 886 pediatric athletes, including 117 with ER, found no major cardiovascular or arrhythmic events over 4.2 years. (Halasz et al. 2021) Additionally, 210 soccer players with 86 having ER and high‐intensity training experienced no cardiovascular events over 5 years. (Peidro et al. 2014) Lastly, a 10‐year follow‐up of 503 athletes with 30% having ER also reported no cases of SCD or symptomatic ventricular tachyarrhythmias. These studies suggest that ER in athletes does not appear to increase the risk of severe cardiovascular events (Junttila et al. 2011).

A study of 122 male youth soccer players in Belgium, with a mean age of 13.5 ± 2.7 years, found no significant difference in family history of sudden cardiac death (SCD) between those with early repolarization (ER) (6.8%) and those without ER (5.2%) (*p* = 0.713). (De Asmundis et al. 2014) Similarly, among 117 rowers, including 35 with ER, none had a family history of sudden death before age 45. (Konopka et al. 2016) In a larger study of 2090 athletes, only 16 subjects (1%) had a family history of sudden cardiac death (SCD) in their first‐degree relatives. None of these 16 subjects' ECGs showed abnormalities, and none had died after at least 3 years of follow‐up. (Claessen et al. 2020) Similarly, Ahmed H. et al. reported no significant difference in the family history of sudden death for individuals with ER (*p* = 0.5).

Although further studies are warranted to fully elucidate the mechanisms and prognostic implications of ER in competitive athletes, to date, no data support an association between inferior ER and SCA in this population. Based on current evidence, all patterns of early repolarization, when observed in isolation and without accompanying clinical markers of pathology, should be considered benign variants in athletes. Further research is essential to confirm these findings and refine risk stratification in this group (Quattrini et al. 2014).

### 4.5 Could ER Be Considered a Vagal Tone ECG Change?

It is suggested that in athletes increased vagal tone from training leads to regional differences in repolarization (Yan et al. 2003), manifesting as ER on ECG. (Corrado et al. 2010; Noseworthy et al. 2011) Specifically, the STE seen in ER likely results from a gradient in transmural repolarization caused by a vagally mediated inward potassium current during the action potential's plateau phase. (Antzelevitch and Yan 2010) This connection to vagal activity explains the benign nature of the ER pattern, as high vagal tone protects against malignant arrhythmias. During intense training, 37% of college athletes showed ER, which increased to 53% after 90 days (*p* < 0.01). ER prevalence rose in endurance athletes like rowers but remained unchanged in strength athletes, like football players, indicating endurance training as a key trigger for ER (Noseworthy et al. 2011).

ER is frequently associated with bradycardia, supporting its protective role. Noseworthy et al. found ER linked to a slower heart rate (odds ratio, 1.54; 95% CI, 1.26–1.87; *p* < 0.001), and Crouse et al. reported sinus bradycardia in 9.1% of their athlete cohort. Brosnan et al. also observed a higher prevalence of sinus bradycardia among elite athletes. Aagaard et al. demonstrated that individuals with ER had a lower resting heart rate (56 ± 8 bpm) compared to those without ER (69 ± 9 bpm, *p* = 0.02). Similarly, Konopka et al. found athletes with ER had significantly lower resting heart rates (58.7 ± 11.3 bpm vs. 65.4 ± 11.9 bpm, *p* < 0.01). Additionally, some research suggests structural cardiac changes in ER athletes compared with non‐ER athletes (Zimmermann et al. 2022).

During and after intense exercise, ER usually disappears due to decreased vagal tone and increased sympathetic activity. However, ER remains even with full autonomic blockade by atropine and propranolol, suggesting other contributing factors. (Macfarlane et al. 2012) A study found that individuals with certain spinal cord injuries, which disrupt central sympathetic regulation, are more likely to experience ER, implying that either increased vagal tone or reduced sympathetic tone may cause the ST elevation associated with ER. (Marcus et al. 2002) Another study showed a higher prevalence of both benign and malignant ER patterns on ECGs immediately after intense exercise in competitive endurance athletes. (Noseworthy et al. 2011) Interestingly, over 50% of healthy elite athletes saw their ER patterns disappear after ceasing training (Serra‐Grima et al. 2015), indicating that ER is a dynamic phenomenon influenced by physical activity levels. In contrast, research by Femke M.A.P. Claessen and colleagues found no increase in ER among strength athletes, such as American football players, suggesting that ER is more likely to occur following endurance rather than strength training. Furthermore, there was no clear link between ER and sports disciplines based on static or dynamic intensity, possibly due to recall or reporting biases in sports participation. (Claessen et al. 2020) Aagaard et al. found that 82% of athletes displayed ECG changes associated with “athlete's heart,” including sinus bradycardia (61%) and ER (32%).

### 4.6 ER And Male Gender

Since the initial study by Pelliccia et al., it has been observed that ER is more common in males. Brosnan et al. found that ER is significantly less frequent in female athletes compared with males (28.7% vs. 47.6%, *p* < 0.0001), and Konopka et al. similarly found a higher prevalence in males (21.36%) than females (8.54%, *p* = 0.01). In this review, studies focusing solely on male athletes consistently reported a high prevalence of ER. This disparity has led to the theory that androgens, particularly higher testosterone levels in adult men, may account for the gender differences in ventricular repolarization due to the profound impact of androgen hormones. (Bidoggia et al. 2000) However, Surawicz et al. found similar ER prevalence in both castrated men and virilized women, suggesting that the relationship between androgens and ER might be more complex than initially thought (Surawicz and Parikh 2002).

### 4.7 Study Limitations

The absence of reported significant cardiovascular events in the studies we reviewed restricts our ability to evaluate the clinical implications of ER in athletes. Additionally, many of the studies included in our review were retrospective or cross‐sectional, which poses inherent limitations. These study designs restrict our ability to follow‐up with participants over time and assess long‐term cardiovascular outcomes, making it difficult to determine the potential risk of future adverse events, such as SCD. The reliance on cross‐sectional data also introduces the possibility of selection bias. Lastly, due to the variability in the methods and populations studied, there may be some degree of heterogeneity in the findings, which limits the generalizability of our results. Future prospective, longitudinal studies with larger sample sizes and standardized methodologies are needed to better understand the prevalence, clinical significance, and potential risks associated with ER in athletes, particularly in relation to SCD and other adverse cardiovascular events.

## 5 Conclusion

ER is a prevalent electrocardiographic pattern among athletes, particularly in male and endurance athletes. Current studies do not establish a direct association between ER and increased mortality, highlighting the need for further research to refine management strategies and establish more precise risk criteria.

## Conflicts of Interest

The author declares no conflicts of interest.

## Data Availability

Data sharing is not applicable to this article as no new data were created or analyzed in this study.
